# Driving Mode Selection through SSVEP-Based BCI and Energy Consumption Analysis

**DOI:** 10.3390/s22155631

**Published:** 2022-07-28

**Authors:** Juai Wu, Zhenyu Wang, Tianheng Xu, Chengyang Sun

**Affiliations:** 1 College of Automation & College of Artificial Intelligence, Nanjing University of Posts and Telecommunications, Nanjing 210023, China; wujuai@njupt.edu.cn (J.W.); scy_njupt@foxmail.com (C.S.); 2 Shanghai Advanced Research Institute, Chinese Academy of Sciences, Shanghai 201210, China; xuth@sari.ac.cn

**Keywords:** brain–computer interface (BCI), steady-state visual-evoked potential (SSVEP), brain-controlled driving mode selection

## Abstract

Background: The brain–computer interface (BCI) is a highly cross-discipline technology and its successful application in various domains has received increasing attention. However, the BCI-enabled automobile industry is has been comparatively less investigated. In particular, there are currently no studies focusing on brain-controlled driving mode selection. Specifically, different driving modes indicate different driving styles which can be selected according to the road condition or the preference of individual drivers. Methods: In this paper, a steady-state visual-evoked potential (SSVEP)-based driving mode selection system is proposed. Upon this system, drivers can select the intended driving modes by only gazing at the corresponding SSVEP stimuli. A novel EEG processing algorithm named inter-trial distance minimization analysis (ITDMA) is proposed to enhance SSVEP detection. Both offline and real-time experiments were carried out to validate the effectiveness of the proposed system. Conclusion: The results show that a high selection accuracy of up to 92.3% can be realized, although this depends on the specific choice of flickering duration, the number of EEG channels, and the number of training signals. Additionally, energy consumption is investigated in terms of which the proposed brain-controlled system considerably differs from a traditional driving mode selection system, and the main reason is shown to be the existence of a detection error.

## 1. Introduction

Brain–computer interfaces (BCIs) are a specific type of communication system between the human brain and the outside world. Generally, BCIs work through capturing and analyzing brain signals [1,2,3]. Recently, tremendous progress has been made in exploring the ability of BCIs. BCI-based voice synthesis [4] and mental handwriting [5] have been proposed with promising performances. High-density and high-resolution recording electrode array, combined with sophisticated signal processing methods such as artificial intelligence, form a powerful tool for realizing a BCI. Depending on the recording devices being invasive or noninvasive, BCIs can also be divided into invasive [4,5] and noninvasive ones [1,2]. Although invasive devices provide a comparatively higher precision, considering that they also can incur a much higher operation danger and maintenance cost, noninvasive devices are utilized in most academic studies and practical applications.

Currently, BCIs mainly serve as a rehabilitation tool for the patients suffering from neurological diseases or disabilities who cannot speak or move [6,7,8,9]. For example, BCI-based high-speed mind-spellers [9] are developed to restore normal communications. Mind-controlled mechanical arms and wheelchairs [6,7] are developed to restore normal activities. However, considering the rapid development of related devices and algorithms, it is believed that BCIs can progress far beyond the current status. BCIs should be applied to a much wider variety of domains, and more groups of people should benefit from them. Early attempts have already started to emerge. BCIs have been applied to robot control [10], smart home [11], disaster management [12], and multimedia interactions [13]. For example, in [14], the authors proposed to use a P300-based BCI to switch various household appliances on and off. They delicately tuned the detection procedure so that intentional control and non-control states can be classified with high precision. In [15], a shared controller was developed to enhance the performance of a BCI-based wheelchair robot, and the automatic control of the robot itself was intelligently combined with brain-actuated control. In [16], a 2D computer cursor control system was built on top of a wireless BCI, and the cursor velocity was controlled with the imagery of hand movement. Comparatively, in [17], the authors proposed a hybrid BCI to enable cursor movement and clicking separately. A motor imagery-based BCI was utilized for cursor movement and a P300-based BCI was utilized for further clicking.

In addition to the domains introduced above, great attention has recently been paid to applying BCI to smart vehicles from both academia and industry. Various prototype systems are developed with the aim of having a more convenient and simpler driving experience. In contrast to autonomous vehicles or unmanned vehicles, BCI-enabled smart vehicles acquire the steering or control commands from drivers through a BCI channel. For example, in [18], the authors presented a paradigm car-control system using a motor imagery-based BCI. Two distinct motor imagery tasks were performed and a shared car control strategy with five different motions was realized. In [19], a steady-state visual-evoked potential (SSVEP)-based BCI was applied to assist remote driving. To evaluate the proposed system, 61 subjects were recruited and a mobile robotic car steering experiment was conducted. The results show that all 61 subjects were able to finish the experiment within a short period of time. In [20], a steering assistant system was developed by showing drivers a guess of the intended turning direction and determining the presence of error-related potentials in their electroencephalo-graph (EEG) signals, i.e., the presence of error-related potentials implies that the prior guess is incorrect and the absence of error-related potentials implies that the prior guess is correct. Additionally, the literature also investigated brain-controlled vehicles from the perspective of action and brake detection [21,22,23], lateral and longitudinal control [24], a collaborative control system incorporating computer vision and radar, etc. In addition, a comprehensive review is given in [25], where brain-controlled vehicles and aerial vehicles are introduced in details. However, research on brain-controlled vehicles is still in its early stages and many important problems remain unsolved, among which we find brain-controlled driving mode selection.

Different driving modes imply different driving characters of the vehicle. Generally, different driving modes are demanded in different road conditions and by different drivers with different driving preferences. Representative driving modes delivered by the automobile manufacturers currently include the economical mode, comfort mode, sport mode, etc., respectively, with a different driving experience and different energy consumption. Specifically, the sport mode has the most aggressive acceleration and consequently the highest energy consumption. Conventional cars only enable driving mode selection through an interactive panel. This requires that drivers use one hand to scroll and select, which turns out to be distracting and cumbersome. To resolve this problem, in this paper, we propose a brain-controlled driving mode selection system based on SSVEP-BCI. The main contributions of this paper can be concluded as follows. Firstly, different driving modes are controlled by setting a damping coefficient to different values. The damping coefficient is designed to rescale the acceleration commands sent by the drivers. Secondly, drivers select the intended driving mode through gazing at a group of visual stimuli, each of which represents a specific mode. Thirdly, the energy consumption of the proposed driving mode selection system is analyzed considering the inevitable detection loss of a BCI.

The remainder of this paper is organized as follows. In Section 2, we introduce the materials and methods, and in greater detail, the SSVEP-based BCI, the driving mode control logic and the energy consumption analysis. In Section 3, the results are presented which include the SSVEP detection accuracy and the energy consumption. Conclusions are drawn in Section 4.

## 2. Materials and Methods

In this section, three aspects of materials and methods are introduced for the proposed brain-controlled driving mode selection system, namely the SSVEP-based driving mode selection, SSVEP detection algorithm and energy consumption analysis. These are separately introduced in the three following subsections.

### 2.1. SSVEP-Based Driving Mode Selection

**Figure 1:**
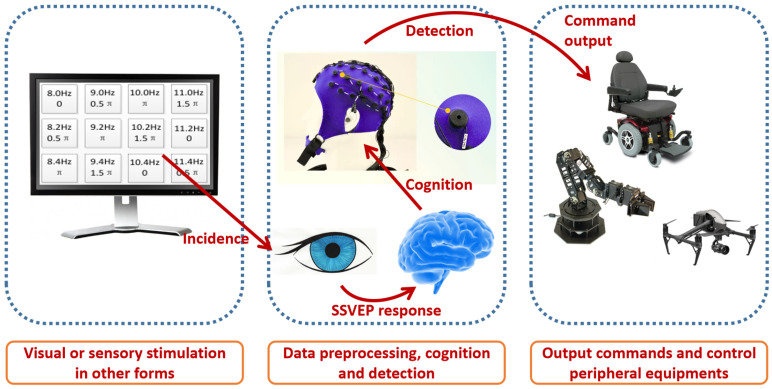
Basic work flow of the passive SSVEP-based BCI system.

There are several technical means to realize a BCI. Different technical means are generally classified by the specific type of brain signal being utilized to develop a BCI. These include motor imagery potential, event-related potential, SSVEP, etc. Among them, SSVEP-based BCI achieves by far the highest information transfer rate due to its highest signal-to-noise ratio and its well-understood signal structure. Generally, SSVEP is elicited when the subjects are subject to repetitive and periodic light stimulation [26,27,28]. SSVEP consists of different order harmonics with the fundamental frequency being the stimulation frequency. In addition, SSVEP has a nonuniform power distribution over scalp. The stronger response of SSVEP is elicited in the occipital region since that is where the visual cortex is located. A basic paradigm of SSVEP-based BCI can be developed as shown in Figure 1. A display screen is needed for stimulation purposes, on which multiple flickering visual targets with distinct frequencies and phases are presented in different positions. Each of the visual targets represents a different control command, whereas the specific mapping varies for different applications. The subjects or the users output the intended commands through gazing at the corresponding targets for a short period of time. Consequently, SSVEP can be elicited and correct classification can be made by recording and processing the EEG signals. After that, commands can be further passed to peripheral equipment to realize certain functionalities, such as wheelchair steering and mechanical arm control, as shown in Figure 1.

**Figure 2:**
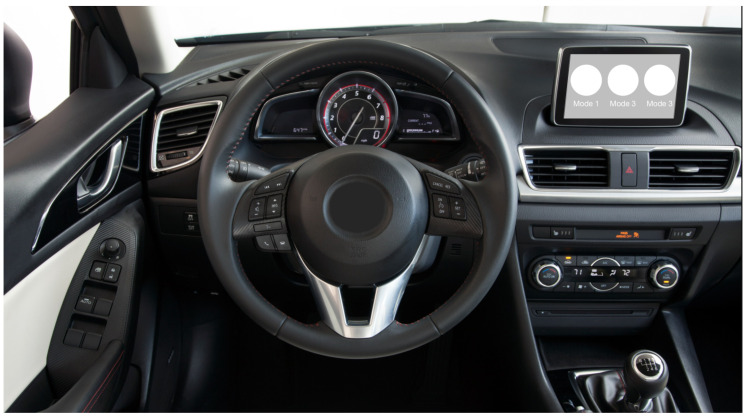
SSVEP-based driving mode selection system: a view of the car dashboard and the SSVEP interface in the middle.

As introduced above, BCI or brain-controlled interaction is a prospective substitute for conventional manual approaches. Furthermore, SSVEP serves as a basic BCI paradigm for various applications. In this paper, we try to develop a brain-controlled driving mode selection system also based on SSVEP. The system design and the control logic are straightforward. Specifically, a part of the central console is utilized for stimuli presentation, as shown in Figure 2. The number of flickering targets is chosen to be the number of different driving modes. Each target represents a different driving mode, which for example, can be economical mode, comfort mode, or sport mode. An EEG machine is needed to closely monitor the brain response of the driver. Signal processing and SSVEP detection algorithms are embedded in the automotive control system to facilitate the whole selection procedure. The detection algorithm will be introduced in the following subsection. To summarize, the proposed system enables drivers to switch between different driving modes by adjusting their eyesight without raising their hands from the steering wheel, thus achieving higher control convenience and efficiency.

### 2.2. SSVEP Detection Algorithm

In the proposed system, accurate driving mode selection relies on accurate SSVEP detection. The choice of SSVEP detection algorithm has a great impact on the detection accuracy and consequently the drive mode selection accuracy that can be achieved. Various detection algorithms have been proposed alongside the development of SSVEP-based BCIs over the last two decades [29]. Among them, the inter-trial distance-minimization analysis (ITDMA) algorithm showed compelling performance and is therefore in this paper for SSVEP detection [30].

ITDMA is a training-based detection algorithm which means that before the real-time usage of the proposed driving mode selection system, training data need to be recorded as template signals so that individualized calibration can be realized. We consider such an SSVEP-based driving mode selection system with $N_{f}$ different driving modes, which means that the number of SSVEP targets is also $N_{f}$. Let $\Gamma =\{\Gamma_{1},\Gamma_{2},\ldots ,\Gamma_{N_{f}}\}$ denote the training set, in which each $\Gamma_{i}$ represents a training set corresponding to the *i*th SSVEP target. Specifically, $\Gamma_{i}=\left\{\Gamma_{i1},\Gamma_{i2},\ldots ,\Gamma_{iN_{t}}\right\}$, and each $\Gamma_{im}$ in $\Gamma_{i}$ is a training signal trial, being a real matrix of dimension $N_{c}\times T$, i.e., $\Gamma_{im}\in R^{N_{c}\times T}$. Each $\Gamma_{im}$ is recorded when the subjects focus their eyes on the *i*th SSVEP target. $N_{c}$ is the number of channels or the number of the recording electrodes. *T* is the number of time sampling points. Furthermore, $N_{t}$ is the number of training trials. In addition, it should be noticed that in SSVEP-based BCIs, training is mostly carried out independently for each different subject, and so is the case in ITDMA.

Generally, the recorded EEG signal during task engagement comprises two basic signal elements, i.e., the task-related SSVEP element $s\left(t\right)\in R$ and the task-unrelated noise element $n\left(t\right)\in R$. Denote the recorded multichannel EEG signal with $x\left(t\right)\in R^{N_{c}}$, and a linear model is utilized to depict the relation between $x\left(t\right)$ and $s\left(t\right)$ and $n\left(t\right)$. *t* is the sampling time index. The model is shown to be:

(1) $$x_{j}\left(t\right)=r_{1,j}\cdot s\left(t\right)+r_{2,j}\cdot n\left(t\right),\;j=1,\;2,\;\ldots ,\;N_{c},$$

where *j* is the channel index, and $x_{j}\left(t\right)$ is the *j*th element of $x\left(t\right)$, i.e., the single-channel EEG signal observed in the *j*th channel. $r_{1,j}$ and $r_{2,j}$ are the mixing coefficients corresponding to $s\left(t\right)$ and $n\left(t\right)$, showing how much they, respectively, contribute to $x\left(t\right)$.

In order to better recover the task-related SSVEP element $s\left(t\right)$, a combination vector $\omega \in R^{N_{c}}$ is utilized to sum up the observed EEG signals from different channels, which is shown as:

(2) $$\hat{s}\left(t\right)=\left(\sum_{j=1}^{N_{c}}\omega_{j}r_{1,j}\right)\cdot s\left(t\right)+\left(\sum_{j=1}^{N_{c}}\omega_{j}r_{2,j}\right)\cdot n\left(t\right).$$

The combination vector ω is also called a spatial filter in the context of SSVEP detection. Ideally, a well-tuned spatial filter ω leads to $\sum_{j=1}^{N_{c}}\omega_{j}r_{1,j}=1$ and $\sum_{j=1}^{N_{c}}\omega_{j}r_{2,j}=0$, and thus we have $\hat{s}\left(t\right)=s\left(t\right)$, i.e., the SSVEP element $s\left(t\right)$ is perfectly recovered. However, the equation system $\sum_{j=1}^{N_{c}}\omega_{j}r_{1,j}=1$ and $\sum_{j=1}^{N_{c}}\omega_{j}r_{2,j}=0$ is overdetermined, which means such an ideal solution of ω does not practically exist, not to mention the difficulty brought by the fact that the mixing coefficients $r_{1,j}$ and $r_{2,j}$ are also not known in advance. To circumvent this problem, existing algorithms proposed to define their own criterion for optimal spatial filters and solve the corresponding optimization problems. The optimality criterion varies among different algorithms. Those algorithms range from task-related component analysis (TRCA) [31], correlated component analysis (CORCA) [32], sum of squared correlation analysis (SSCOR) [33], etc.

In the proposed driving mode selection system, SSVEP detection is carried out utilizing the ITDMA algorithm [30]. In short, ITDMA calculates the spatial filters through minimizing the inter-trial distance over the training set. Specifically, for the *i*th target, the inter-trial distance over the training set $\Gamma_{i}$ is defined as:

(3) $$\sum_{m_{1}=1}^{N_{t}}\sum_{m_{2}=1,m_{2}\neq m_{1}}^{N_{t}}{∥\omega^{\prime }\Gamma_{im_{1}}-\omega^{\prime }\Gamma_{im_{2}}∥}_{2}^{2},$$

where ${∥\cdot ∥}_{2}^{2}$ calculates the square of the Euclidean norm and $\omega^{\prime }$ is the transpose of ω. The Formula (3) is adopted as the objective function that the ITDMA algorithm tries to minimize. In addition, it should be noticed that the spatial filters are calculated by ITDMA in a target-independent fashion, i.e., the spatial filter ω obtained by minimizing (3) is actually the spatial filter $\omega^{\left(i\right)}$ designated for only the *i*th target. However, minimizing (3) with no constraints is mathematically not a well-defined optimization problem and always produces a zero solution. Therefore, a regularization condition is considered here, which is shown as:

(4) $$\sum_{m=1}^{N_{t}}\sum_{j_{1}=1}^{N_{c}}\sum_{j_{2}=1}^{N_{c}}\omega_{j_{1}}\omega_{j_{2}}\mathrm{Cov}\left(\Gamma_{im}\left(j_{1},t\right),\Gamma_{im}\left(j_{2},t\right)\right)=1.$$

Literally, this regularization condition implies that the integrated training signal is of unity power. The integrate training signal is formed by concatenating the $N_{t}$ trials in $\Gamma_{i}$ in series. Then, the approach ITDMA adopts to obtain the spatial filter is to minimize (3) conditioned on (4). Denote $P=\sum_{m_{1}=1}^{N_{t}}\sum_{m_{2}=1,m_{2}\neq m_{1}}^{N_{t}}\Gamma^{\left(m_{1},m_{2}\right)}\Gamma^{\left(m_{1},m_{2}\right)\prime }$ with $\Gamma^{\left(m_{1},m_{2}\right)}=\Gamma_{im_{1}}-\Gamma_{im_{2}}$, and $Q\in R^{N_{c}\times N_{c}}$ being a square matrix with $Q\left(j_{1},j_{2}\right)=\sum_{m=1}^{N_{t}}\mathrm{Cov}\left(\Gamma_{im}\left(j_{1},t\right),\Gamma_{im}\left(j_{2},t\right)\right)$, and the optimization problem stated above can be further simplified as:

(5) $$\begin{array}{cc} \; & \min_{\omega }\;\omega^{\prime }P\omega ,\\ \; & \;s.t.\;\omega^{\prime }Q\omega =1, \end{array}$$

which is actually a common constrained optimization problem and the optimum spatial filter $\omega^{\left(i\right)}$ is given by the eigenvector corresponding to the minimum eigenvalue of matrix $Q^{-1}P$.

As introduced above, since the ITDMA algorithm calculates the spatial filters in a target-independent way, $N_{f}$ spatial filters can be obtained in the training stage, i.e., $\omega^{\left(1\right)}$, $\omega^{\left(2\right)}$, … and $\omega^{\left(N_{f}\right)}$. Considering the similarity among the $N_{f}$ spatial filters and in order to make full use of them, an ensemble spatial filter E is developed by ITDMA in the test stage and it is given by:

(6) $$E=\left[\omega^{\left(1\right)},\omega^{\left(2\right)},\ldots ,\omega^{\left(N_{f}\right)}\right],$$

where all $N_{f}$ spatial filters are taken into account. During the test stage, the task is to decide by which SSVEP target a new test signal $S\in R^{N_{c}\times T}$ is elicited by. The decision rule taken by ITDMA is shown to be:

(7) $$\pi =\arg\min_{i=1}^{N_{f}}\;{∥E^{\prime }S-E^{\prime }\bar{\Gamma_{i}}∥}_{F},$$

where ${∥\cdot ∥}_{F}$ calculates the Frobenius norm, and the template signal $\bar{\Gamma_{i}}$ is obtained by averaging all $N_{t}$ signal trials in the training set $\Gamma_{i}$, which is shown as

(8) $$\bar{\Gamma_{i}}=\frac{1}{N_{t}}\sum_{m=1}^{N_{t}}\Gamma_{im}.$$

**Figure 3:**
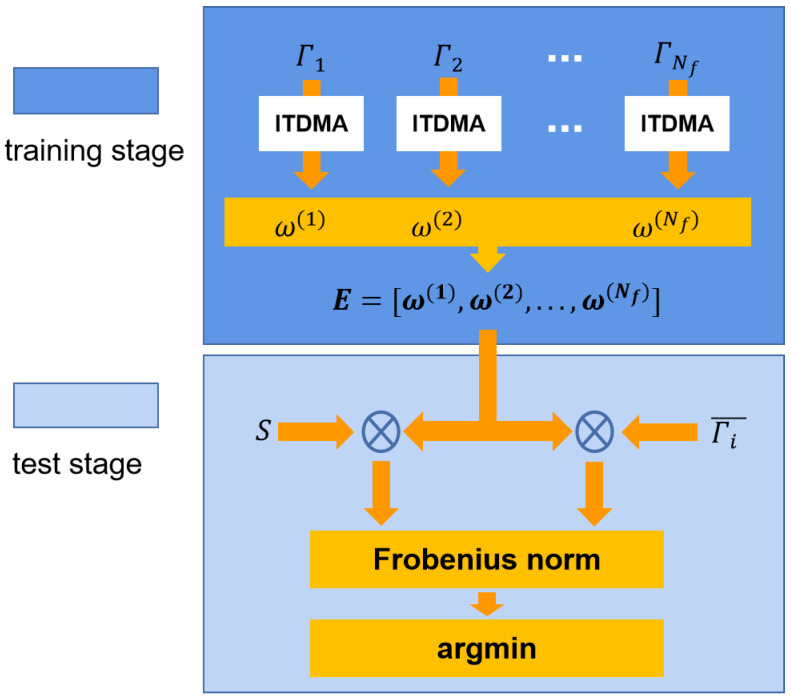
Basic workflow of ITDMA for SSVEP detection.

In short, upon the ensemble spatial filter E, the target which achieves the minimum distance between the spatial-filtered test signal and template signal is decided to be the detection result. The basic workflow of ITDMA is presented in Figure 3, where the whole detection process of ITDMA is divided into two separate parts, namely the training stage and the test state. Additionally, it should be noticed that in contrast with existing SSVEP detection algorithms such as TRCA, CORCA and SSCOR which take correlation values as their algorithmic metric in both the training stage and the test stage, ITDMA takes the distance metric instead, and that is also where ITDMA attains its name.

### 2.3. Energy Consumption Analysis

The selection of the driving mode has a direct impact on the energy consumption of vehicles. Intuitively, the more aggressive the driving mode is, the higher energy consumption can be incurred. In [34], the authors investigated the relation between energy consumption and driving characters, and developed an oil consumption model. In [35], the authors investigated the impact of driving characteristics on an electric vehicle energy consumption and range. The results they achieved showed that energy consumption is considerably affected by driving style, and a difference in energy consumption up to 30% can be made between the moderate driving manner and aggressive driving manner. In [36], the authors tried to build a black-box model to describe the relationship between energy consumption and factors such as velocity, road condition and acceleration, and based on this model, driving modes can be optimized so that more energy can be saved.

We consider an electric car. The instantaneous energy consumption can be derived through analyzing the automobile kinetics. Specifically, according to Newton’s laws of motion, the kinetic equation is shown as:

(9) $$F=mgf+\delta m\frac{dv}{dt}+\frac{\rho C_{D}A}{2}v^{2},$$

where *F* is the driving force, *m* is the mass of vehicle, *f* is the rolling friction factor, δ is the scaling factor of vehicle mass, ρ is the air density, $C_{D}$ is the aerodynamic drag coefficient, *A* is the frontal area, *v* is vehicle velocity, and *t* is time. Generally, we choose ρ = 1.2258 kg/m${}^{3}$. It should be noticed that Equation (9) assumes that the urban road is of high quality. To calculate the energy consumption, we discretize Equation (9) as:

(10) $$F_{t}=mgf+\delta m\frac{v_{t+1}-v_{t}}{\Delta t}+\frac{\rho C_{D}A}{2}{v_{t}}^{2},$$

where *t* is now the discrete time index and Δt is the time interval. According to Equation (10), the energy consumption can be calculated as:

(11) $$E_{t}=\sum_{t=1}^{T}F_{t}\times v_{t}\times \Delta t,$$

where the energy consumed in each discrete time interval is computed and summed up. *T* is the ending time.

We try to characterize the influence of different driving modes on the energy consumption of vehicles. Different driving modes are actually different transfer functions between an automotive response such as acceleration and deceleration, and the actual operations of drivers. Take the three aforementioned driving modes, for example, i.e., the economical mode, comfort mode and sport mode; among these, the sport mode tends to more aggressively transfer the operation commands of drivers, particularly during the startup and the acceleration stage. We considered a simplified mathematical model to describe the different driving modes focusing on the startup and the acceleration stage, which is given as:

(12) $$a=a_{ideal}+\left(1-\lambda \right)a_{user},$$

where $a_{ideal}$ is the ideal recommended acceleration, $a_{user}$ is the acceleration command sent from the driver, *a* is the actual acceleration, and λ is the acceleration damping coefficient with a positive value between 0 and 1. According to Equation (12), different driving modes can be realized by setting the damping coefficient λ to different values. Generally, a smaller choice of λ implies a more aggressive and more active driving mode. For example, if λ=1, then the acceleration is constant and always equals the recommended value for the sake of energy efficiency; however, the driving experience is to a certain extent compromised.

To evaluate the influence of different driving modes on energy consumption, three experienced drivers were recruited to participate in a simulated driving experiment with simulation software. In the experiment, the three drivers were required to drive the same car in closed roads for 5 km. Along the route, except for the two crossroads where the drivers needed to brake and restart the car, they drove at a constant speed. Some of the related parameters are shown in Table 1. These parameters are chosen according to [37]. The vehicle velocity was recorded during the whole experiment, and then the energy consumption can be calculated considering Equation (11) together with the vehicular power amplification module.

**Figure 4:**
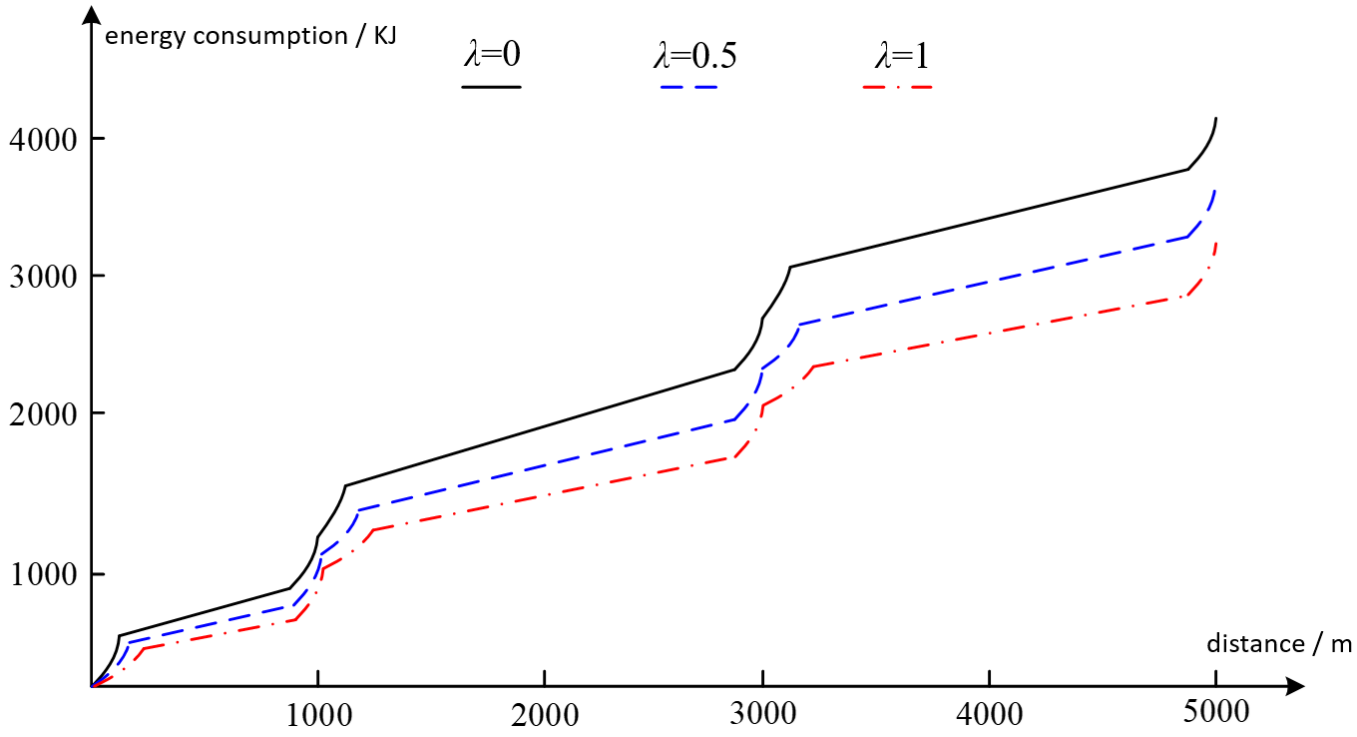
Energy consumption versus driving distance.

The results are shown in Figure 4 with the horizontal axis being the driving distance and the vertical axis being the energy consumption. For each driver, three experiment trails were completed independently. In each experiment trial, the damping coefficient λ was set to a different value, i.e., λ=0, λ=0.5, λ=1, corresponding to three different driving modes. As shown in Figure 4, the results are derived by averaging over the three drivers. It can be seen that the energy consumption increases as the driving distance varies from 0 to 5000 m. In addition, two jumps of energy consumption can be found in Figure 4 at 1000 m and 3000 m distance, respectively, corresponding to two crossroads where the drivers needed to brake and restart the vehicle, for which considerable energy was consumed. When λ=0, the total energy consumed to finish the drive is 4105 KJ. When λ=0.5, the value is 3618 KJ. When λ=1, the value is 3206 KJ. The results indicate that a smaller choice of damping coefficient λ or a more aggressive driving mode causes higher energy consumption.

## 3. Results and Discussions

### 3.1. Experiment Settings

#### 3.1.1. SSVEP interface

In this section, we provide the experiment results about the proposed SSVEP-based driving model selection system. A prototype test system was developed exactly as shown in Figure 2. A portable screen was utilized for visual-stimuli display. The screen is placed in the middle of the central console. Three different driving modes are considered here, which means that three SSVEP targets are realized to represent them. The three driving modes are differentiated by the damping coefficient λ introduced in the final section. Three different values of λ were chosen to be λ=0, λ=0.5, and λ=1. As for the corresponding SSVEP targets, the flickering frequencies are chosen to be 10 Hz, 12 Hz, and 14 Hz. A synchronized SSVEP system is adopted in the experiment, which means that periodically, a time stamp is generated and all three SSVEP targets start to flicker and last for a short period of time. During this flickering stage, the drivers select the intended driving mode through gazing at the corresponding SSVEP target in a synchronized manner. In the experiment, the time stamp arrives every 5 s, which means that every 5 s, a driving mode selection command can be generated and output. The 5 s of time is further divided into two parts, with the first being the flickering stage as introduced above, and the second being the rest stage. The rest stage is used to separate two consecutive flickering stages, allowing the drivers to have some rest and minimize the interference between two consecutive selection commands. In the experiment, the duration of the flickering stage is set to 1.5 s, which means that the duration of the rest stage is 3.5 s.

#### 3.1.2. Offline and Real-Time Driving Mode Selection

Three drivers took part in the experiment. The experiment was comprised of two parts, i.e., the offline experiment and the real-time experiment. In the offline experiment, simulated driving mode selection was conducted. The drivers sat in the driving seat and switched between the three driving modes as constructed by a predetermined order. The EEG signal was recorded and processed, and the selection accuracy was calculated by comparing the detection results and the predetermined order in an offline manner. Comparatively, in the real-time experiment, similarly to the prior experiment introduced in the last section, the drivers were also required to finish a simulated drive of 5 km in closed road. The driving mode was selected using the proposed SSVEP-based selection system at the beginning and remained unchanged until the end of the 5 km drive. It should be noticed that the actual driving mode during the 5 km drive might possibly be the intended mode of the driver, but it could also not be the intended one due to detection errors. During the real-time experiment, the vehicle status was also recorded and energy consumption was estimated. The offline experiment was repeated 20 times, and the real-time experiment was repeated 5 times.

#### 3.1.3. EEG Recording and Preprocessing

During the experiment, the drivers wore an electrode cap to capture EEG and signals were recorded using a Synamps2 system (Neuroscan, Inc., Herndon, VA, USA). The sampling rate was 1000 Hz. The electrodes FPz and Cz (according the international 10–20 system) were chosen, respectively, the ground and the reference. Six active channels near the occipital region were chosen for EEG recording and later detection process. These were POz, PO4, PO6, O1, Oz and O2. To preprocess the EEG signals, the following procedures were adopted. First, raw signals were down-sampled to 250 Hz. Second, a notch filter at 50 Hz and a band-pass filter between 7 and 70 Hz were jointly utilized to remove noise. Third, a visual pathway latency of 135 ms was considered, which means only signals recorded 135 ms after the stimulus onset were used [29]. In addition, in the offline experiment, the EEG signals were recorded and stored and later processed. The detection accuracy was estimated following cross-validation. Comparatively, in the real-time experiment, EEG signals were recorded and immediately processed, and the detection result was subsequently translated to execute driving-mode selection.

#### 3.1.4. Subjects

Six graduate students participated in the experiment (two females, with an average age of 25). All six of them took part in the offline experiment, and only three of them took part in the real-time driving experiment. All subjects had driving licenses. All subjects signed their consent and received financial compensation.

### 3.2. SSVEP Detection Performance

**Figure 5:**
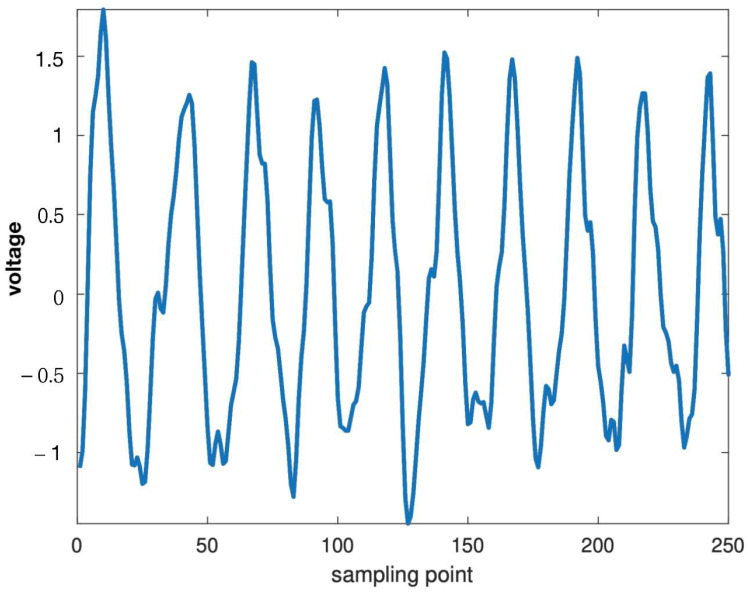
An example of a refined SSVEP signal after denoising, spatial filtering, and trial averaging (10 Hz).

In this subsection, we present the results about SSVEP detection accuracy, i.e., the driving mode selection accuracy of the proposed brain-controlled system. More specifically, we present how the ITDMA algorithm is compared with the benchmark algorithms (TRCA [31] and SSCOR [33]) in terms of SSVEP detection accuracy. Considering that all three algorithms were training-based algorithms, leave-one-out cross-validation was used to evaluate the detection accuracy, which means the 20 signal-trials recorded in the offline experiment take turns to be the test signal with the other 19 signal-trials being training signals. Additionally, the information transfer rate was also evaluated to verify the efficiency of the proposed brain-controlled system [38]. The formula used to calculate the information transfer rate is given by:

(13) $$\begin{array}{c} \mathrm{ITR}=\frac{1}{T_{0}}\cdot \left(\log_{2}N_{f}+P\log_{2}P+\left(1-P\right)\log_{2}\left(\frac{1-P}{N_{f}-1}\right)\right), \end{array}$$

where $N_{f}$ is the number of SSVEP targets, *P* is the detection accuracy and $T_{0}$ is the average time consumed to output one command; the rest time between every two adjacent SSVEP trials was chosen to be 0.5 s. Before diving deep into detection performance analysis, Figure 5 provides an example of refined SSVEP signal after denoising, spatial filtering, and trial averaging (10 Hz stimulation, 250 Hz sample rate, 1 s signal length).

#### 3.2.1. SSVEP Detection Accuracy Versus Flickering Duration

**Figure 6:**
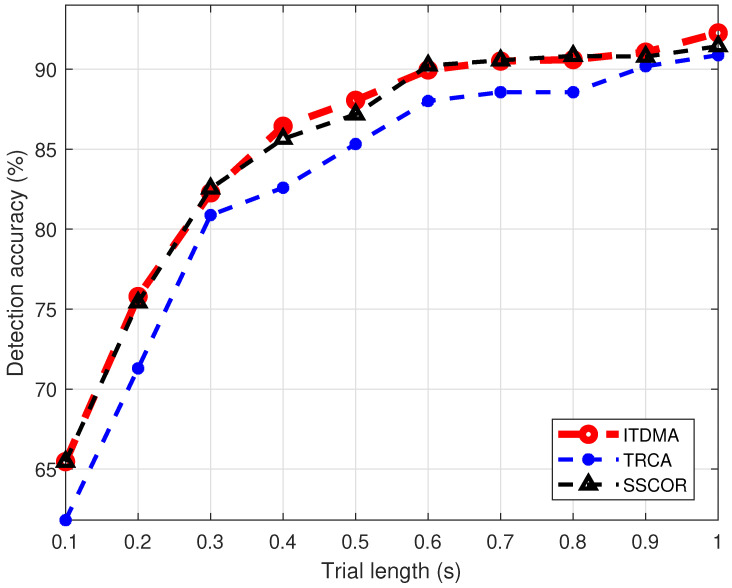
Detection accuracy comparison versus the trial length.

First, we investigated how the SSVEP detection accuracy of ITDMA varies with the duration of the flickering stage. Intuitively, longer stimulation incurs higher the signal-to-noise ratio of SSVEPs and consequently a higher detection accuracy. We analyzed the signals recorded in the offline experiment and obtained the following results. For these results, the detection accuracy is calculated through averaging over the 6 drivers and all 20 repetitions of the offline experiment. The flickering duration is changed from 0.1 s to 1 s with a step of 0.1 s. Six channels and ten training trials were used. The results are shown in Figure 6. It can be seen that the detection accuracy stably rises as the flickering duration is increased from 0.1 s to 1 s. Additionally, ITDMA outperforms both TRCA by a distinct margin and achieves a similar detection performance to that of SSCOR. Specifically, when the flickering duration is 0.4 s, the detection accuracies achieved by ITDMA, TRCA and SSCOR are, respectively, 86.5%, 82.6% and 85.6% (corresponding to the information transfer rate being 0.98 bit/s, 0.83 bit/s and 0.94 bit/s); when the flickering duration is 1 s, the detection accuracies achieved by ITDMA, TRCA and SSCOR are, respectively, 92.3%, 90.9% and 91.4% (corresponding to the information transfer rate being 0.74 bit/s, 0.70 bit/s and 0.72 bit/s). According to these results, an optimal flickering duration can be determined through balancing between the selection latency and selection accuracy. However, this is already out of the scope of this paper.

#### 3.2.2. SSVEP Detection Accuracy Versus the Number of EEG Channels

**Figure 7:**
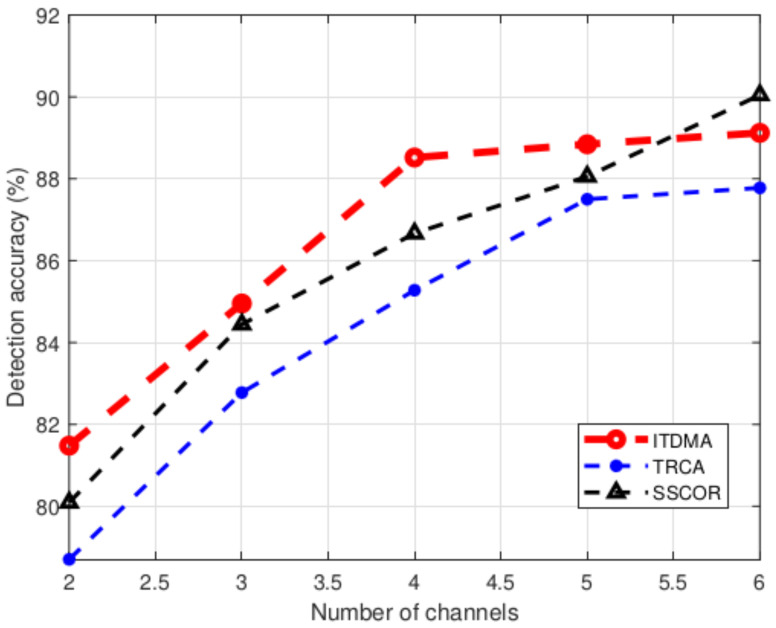
Detection accuracy comparison versus the number of channels.

Second, we investigate how the SSVEP detection accuracy of ITDMA varies with the number of channels recording EEG. Intuitively, recording EEG from more channels implies a higher combination gain can be achieved, and thus a better SSVEP detection performance can be realized. Actually, numerous studies have paid attention to more efficiently combining multiple EEG channels, and thus so does the ITDMA algorithm introduced in this paper. Here, the number of channels is changed from 2 to 6. The channels are sequentially chosen from POz, PO4, PO6, O1, Oz and O2 as introduced above. The flickering duration is 0.5 s. The results are shown in Figure 7. It can be seen that recording EEG from more channels generally results in a higher detection accuracy, although a zigzag relation is presented in Figure 7. Additionally, ITDMA outperforms both TRCA and SSCOR in most cases, except when the number of channels is 6. More specifically, when the number of channels is 2, the detection accuracies achieved by ITDMA, TRCA and SSCOR are, respectively, 81.5%, 78.7% and 80.1% (corresponding to the information transfer rate being 0.71 bit/s, 0.62 bit/s and 0.67 bit/s); when the number of channels is 6, the detection accuracies achieved by ITDMA, TRCA and SSCOR are, respectively, 89.1%, 87.8% and 90.0% (corresponding to the information transfer rate being 0.98 bit/s, 0.93 bit/s and 1.02 bit/s). However, it should be noticed that recording from more channels is also practically more cumbersome with regard to hardware requirement and necessary preparations such as putting on an electrodes cap and injecting gel. Therefore, an optimal number of channels can also be selected through balancing between the detection performance and convenience, although it is also out of the scope of this paper.

#### 3.2.3. SSVEP Detection Accuracy Versus the Number of Training Trials

**Figure 8:**
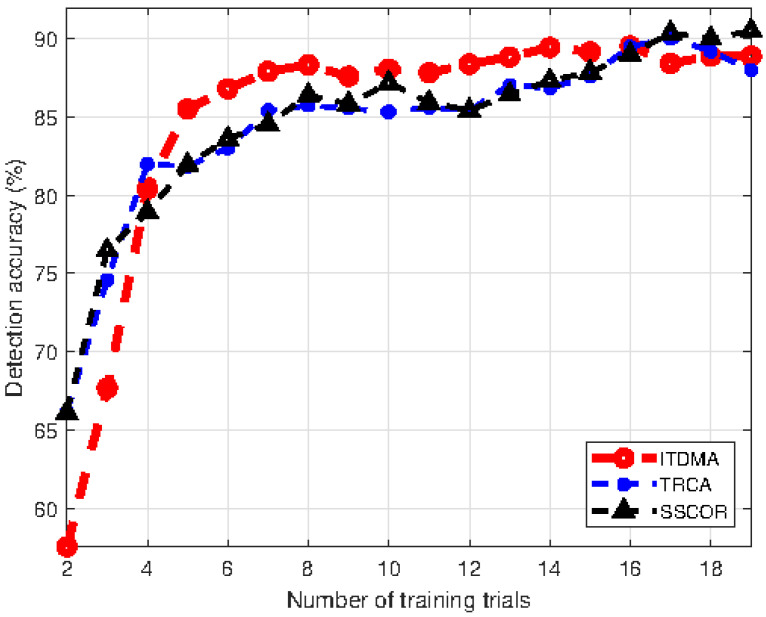
Detection accuracy comparison versus the number of training trials.

Third, we investigated how the SSVEP detection accuracy of ITDMA varies with the number of training trials. Generally, more training data lead to a more accurate detection model. Specifically, as shown in Equation (7), a better spatial filter can be calculated and a template with less noise can be obtained. Here, the number of training trials is changed from 2 to 19, and the results are shown in Figure 8. The number of channels is 6, and the flickering duration is 0.5 s. It can be seen that the detection accuracies achieved by ITDMA, TRCA and SSCOR all generally increase with the number of training trials. Additionally, as for the comparison among the three algorithms, the results shown in Figure 8 are slightly different from those in Figure 6 and Figure 7. Specifically, when the number of training trials is less than 4, the detection accuracy achieved by ITDMA is lower than that achieved by TRCA and SSCOR. Specifically, when the number of training trials is 2, the detection accuracies achieved by ITDMA, TRCA and SSCOR are, respectively, 57.6%, 66.1% and 66.1% (corresponding to the information transfer rate of 0.18 bit/s, 0.32 bit/s and 0.32 bit/s). Comparatively, when the number of training trials is greater than 4, ITDMA generally achieves the best detection performance. For example, when the number of training trials is 10, the detection accuracies achieved by ITDMA, TRCA and SSCOR are, respectively, 88.1%, 85.3% and 87.2% (corresponding to the information transfer rate being 0.94 bit/s, 0.84 bit/s and 0.91 bit/s). ITDMA performs better when there are sufficient training data. Furthermore, it should be noticed that more training data enhance the detection performance, however, the time consumed by the data collection procedure cannot be overlooked, which may considerably complicate the preparation and usage of the proposed system. Therefore, an optimal number of training trials can also be selected through balancing between detection performance and preparation simplicity, although it is also out of the scope of this paper.

### 3.3. Energy Consumption

Here, we present the results about the energy consumption in the real-time experiment. In the real-time experiment, the drivers were required to select the intended driving mode using the proposed SSVEP-based selection system and then finish the 5 km drive. In contrast to the prior experiment introduced in Section 2, the misclassification of SSVEP could happen in the real-time experiment, and the drivers might finish the whole drive in accidentally chosen driving mode. Six EEG channels were used and the number of training trials was five. The flickering duration was set to 0.5 s. The other parameters were also set following Table 1. The results are shown in Table 2. The first row is the intended driving mode, which is specified by the choice of the damping coefficient λ; the second row presents the energy consumption in the real-time experiment; for comparison purposes, the third row presents the corresponding energy consumption in the prior experiment. Similar to the results presented in Figure 4, from Table 2 it can be seen that in the real-time experiment, selecting a different driving mode also results in a different energy consumption. Furthermore, a more aggressive driving mode or a smaller choice of the damping coefficient is chosen, and thus, more energy is consumed. However, the results also show that the real-time experiment differs from the prior experiment in terms of energy consumption, even for the same intended driving mode. The reason is that, in the prior experiment, the driving mode was selected through a traditional interface where the selection error can be completely prevented; however, in the real-time experiment, an SSVEP-based BCI was used and the selection error can occur due to imperfect detection. For example, a driver tries to select the sport mode using the proposed SSVEP-based system. They focus on the corresponding flicker target for a while, and then the SSVEP response is elicited and EEG is recorded. However, it could happen that after processing the EEG signals, the detection algorithm determines that the driving mode intended by the driver is the comfort mode, which makes a selection mistake. Consequently, the energy-consumption characteristics are affected, and difference between the proposed driving mode selection system and the traditional counterpart as shown in Table 2 is produced.

### 3.4. Challenges and Limitations

However, the proposed brain-controlled driving mode selection system still has some challenges and limitations. Firstly, to enable the brain-control functionality, drivers should put on an electrode cap to collect EEG signals from scalp. On some occasions, gel must be injected to enhance conduction, which may be considerably inconvenient. Single-channel EEG recording and processing may be a potential solution to this problem [39]. Additionally, in contrast with laboratory settings, a real driving scenario can cause frequent body motion which may result in serious EEG noise and degenerate detection. Secondly, to elicit a strong SSVEP signal, the proposed system requires drivers to focus on SSVEP targets for a period of time (approximately 1 s), which can distract drivers from road and cause hazardous situations, especially at high driving speeds. Other paradigms such as motor imagery can be adopted to alleviate this problem. Thirdly, the control efficiency has to be further enhanced. New techniques such as implanted electrodes and artificial intelligence-based signal processing algorithms are desired to develop a BCI with a higher information transfer rate so that more complex functionalities can be realized, such as brain-controlled steering. Fourthly, real-time brain-controlled operation during the driving process is needed to further validate the proposed system. A synchronous SSVEP system that supports real-time control at any time is considered as the future work.

## 4. Conclusions

In this paper, we explored applying SSVEP-based BCI to the traditional automobile industry and a brain-controlled driving mode selection system is proposed. Specifically, each driving mode is represented by a different SSVEP target, and drivers can select the intended mode by simply focusing their eyes on the corresponding target. A novel detection algorithm named ITDMA is proposed for EEG signal processing and SSVEP detection. Both offline and real-time experiments were carried out to verify the effectiveness of the proposed system. The results show that a high selection accuracy of up to 92.3% can be realized, although this depends on the specific choice of flickering duration, the number of EEG channels, and the number of training signals. Additionally, the energy consumption was investigated in terms of which the proposed brain-controlled system considerably differs from the traditional driving mode selection system, and the main reason is shown to be the existence of a detection error.

## Figures and Tables

**Table 1:** Parameter settings in the driving experiment.

Parameters	Value
$a_{ideal}$	2 m/s${}^{2}$
$a_{user}$	0.5 m/s${}^{2}$
*m*	1500 kg
*f*	0.015
δ	1.2
$C_{D}$	0.3
*A*	2 m${}^{2}$
*v*	15 m/s

**Table 2:** Energy consumption corresponding to the intended driving mode.

**The Damping Coefficient** λ	0	0.5	1
**Energy Consumption in the Real-Time Experiment (kJ)**	3916	3504	3452
**Energy Consumption in the Prior Experiment (kJ)**	4105	3618	3206

## Data Availability

The data presented in this study are available on request from the corresponding author. The data are not publicly available due to privacy and ethical concerns.

## Abbreviations

The following abbreviations are used in this manuscript:

MDPI	Multidisciplinary Digital Publishing Institute
DOAJ	Directory of Open Access Journals
TLA	Three-letter acronym
LD	Linear dichroism
